# DNA methylation changes in metabolic and immune-regulatory pathways in blood and lymph node CD4 + T cells in response to SIV infections

**DOI:** 10.1186/s13148-020-00971-w

**Published:** 2020-12-09

**Authors:** Simon P. Jochems, Beatrice Jacquelin, Nicolas Tchitchek, Florence Busato, Fabien Pichon, Nicolas Huot, Yi Liu, Mickaël J. Ploquin, Elodie Roché, Rémi Cheynier, Nathalie Dereuddre-Bosquet, Christiane Stahl-Henning, Roger Le Grand, Jorg Tost, Michaela Müller-Trutwin

**Affiliations:** 1 HIV Inflammation and Persistence Unit, Institut Pasteur, 28 Rue Didot, 75015 Paris, France; 2 Sorbonne Paris Cité, Université Paris Diderot, Paris, France; 3 IDMIT Department/IBFJ, Immunology of Viral Infections and Autoimmune Diseases (IMVA), INSERM U1184, CEA, Université Paris Sud, Fontenay-aux-Roses, France; 4 Laboratory for Epigenetics and Environment, Centre National de Recherche en Génomique Humaine, CEA-Institut de Biologie François Jacob, Evry, France; 5 UMR8104, CNRS, U1016, INSERM, Institut Cochin, Université de Paris, 75014 Paris, France; 6 Unit of Infection Models, German Primate Center (DPZ), Göttingen, Germany; 7 Present Address: Leiden University Medical Center, Leiden, The Netherlands

## Abstract

The molecular mechanisms underlying HIV-induced inflammation, which persists even during effective long-term treatment, remain incompletely defined. Here, we studied pathogenic and nonpathogenic simian immunodeficiency virus (SIV) infections in macaques and African green monkeys, respectively. We longitudinally analyzed genome-wide DNA methylation changes in CD4 + T cells from lymph node and blood, using arrays. DNA methylation changes after SIV infection were more pronounced in lymph nodes than blood and already detected in primary infection. Differentially methylated genes in pathogenic SIV infection were enriched for Th1-signaling (e.g., *RUNX3*, *STAT4*, *NFKB1*) and metabolic pathways (e.g., *PRKCZ*). In contrast, nonpathogenic SIVagm infection induced DNA methylation in genes coding for regulatory proteins such as LAG-3, arginase-2, interleukin-21 and interleukin-31. Between 15 and 18% of genes with DNA methylation changes were differentially expressed in CD4 + T cells in vivo. Selected identified sites were validated using bisulfite pyrosequencing in an independent cohort of uninfected, viremic and SIV controller macaques. Altered DNA methylation was confirmed in blood and lymph node CD4 + T cells in viremic macaques but was notably absent from SIV controller macaques. Our study identified key genes differentially methylated already in primary infection and in tissues that could contribute to the persisting metabolic disorders and inflammation in HIV-infected individuals despite effective treatment.

## Introduction

DNA methylation, in concert with other epigenetic mechanisms, has multifaceted effects on the immune system, including T helper lineage fate determination [1], differentiation of memory cells [2], resolution of inflammation [3] and control of cellular proliferation [4]. DNA methylation can influence immune activation and inflammation as demonstrated by the observation that aberrant DNA methylation occurs in autoimmune and inflammatory diseases, such as systemic lupus erythematosus and rheumatoid arthritis [5–7].

HIV and SIV infections in humans and macaques (MAC), respectively, induce a chronic state of immune activation, which is the driving force behind CD4 + T cell depletion and AIDS [8]. Under efficient anti-retroviral treatment (ART), residual inflammation persists in most individuals, increasing the risk of non-AIDS comorbidity and mortality [9]. Despite intense efforts, the molecular mechanisms underlying the persistent inflammation in virologically suppressed people living with HIV (PLWH) remain incompletely characterized [10]. HIV persists in cellular and anatomical reservoirs in virologically suppressed PLWH. Recent studies have elucidated that HIV targets preferentially CD4 + T cells that display a particular metabolic profile with strong glycolytic activity [11, 12].

HIV infection of CD4 + T cells in vitro induces changes in DNA methylation of genes that regulate immune responses (interferon gamma (*IFNG*), interleukin-2 (*IL2*), forkhead box P3 (*FOXP3*) and programmed cell death 1 (*PDCD1*, the gene encoding PD-1)) [13–16]. Two studies in adults and perinatally HIV-infected children demonstrated that HIV infection is associated with changes in DNA methylation, including genes on the HLA locus in chromosome 6 [17, 18]. Another study that compared a pair of twins with discordant HIV infection status showed that several other genes are also differentially methylated, including insulin-like growth factor binding protein 6 (*IGFBP6*) [19]. Previous studies on HIV infection have generally been performed cross-sectionally and analyzed blood but not tissues. A genome-wide, longitudinal analysis of how HIV affects the host methylome in tissue CD4 + T cells is lacking. The study of CD4 + T cells is important because not only they are the main target cell of the virus but also because of their abnormal activation during HIV infection; most CD4 + T cells that are abnormally activated during HIV infection are not infected. Moreover, under current ART regimens, CD4 + T cells show a diminished capacity to restore their normal metabolic function compared to CD8 + T cells, B cells and NK cells [20]. Initiation of ART during acute HIV-1 infection leads to a better control of immune activation than initiation in chronic infection [10]. However, initiation of ART, even at very early stages of infection, does not fully restore all HIV-associated disorders. For instance, ART started in Fiebig stage III of HIV-1 acute infection restores Th17 cell numbers but not their polyfunctionality [21].

African nonhuman primates are the animal reservoir of HIV. These natural hosts of SIV, such as African green monkeys (AGM) and sooty mangabeys, display similar high levels of viral load in blood and gut as untreated PLWH [10, 22–25]. However, natural hosts of SIV do not display chronic immune activation or progression to AIDS [22, 24]. After mounting an acute immune response to SIV during primary infection, natural hosts downregulate the SIV-induced immune activation [24]. This control of immune activation is maintained for their entire life span, suggesting an active, efficient and long-lasting mechanism. Moreover, unlike human or MAC, natural hosts maintain a normal T helper subset balance [10, 26, 27]. Given the robust and long-lasting control of immune activation in the natural hosts, we raise the hypothesis that specific epigenetic modifications contribute to maintaining this long-term control.

In the current study, we compared changes in DNA methylation induced by pathogenic and nonpathogenic SIV infection. We performed a longitudinal analysis to identify DNA methylation modifications induced in response to SIV infection in the two distinct animal models. The DNA methylation patterns were analyzed in a genome-wide manner from purified CD4 + T cells. We analyzed CD4 + T cells not only in blood, but also in a major organ for the education of immune responses and site of viral replication, the lymph nodes (LN). Herein, we searched for differences in DNA methylation profiles between acute and chronic infection. In order to analyze which DNA methylation profiles are associated with uncontrolled viremia and immune activation, we compared viremic MAC to two non-progressive animal models: SIV-infected AGM and SIV controller MAC. SIV controller MAC mimic humans who can spontaneously control HIV [28, 29]. In these three non-human primate models, we investigated whether SIV infection-induced changes in DNA methylation occurred in genes associated with (1) immune activation and regulation; (2) T cell differentiation; (3) anti-viral immune responses such as viral restriction factors; (4) antigen-presentation; (5) cell metabolism; and (6) cell proliferation. The study revealed novel genes regulated by DNA methylation in CD4 + T cells and specific genes differentially methylated during pathogenic and nonpathogenic SIV infection related to modulation of immune response, metabolism and tissue integrity.

## Results

### SIV infections course

**Fig. 1:**
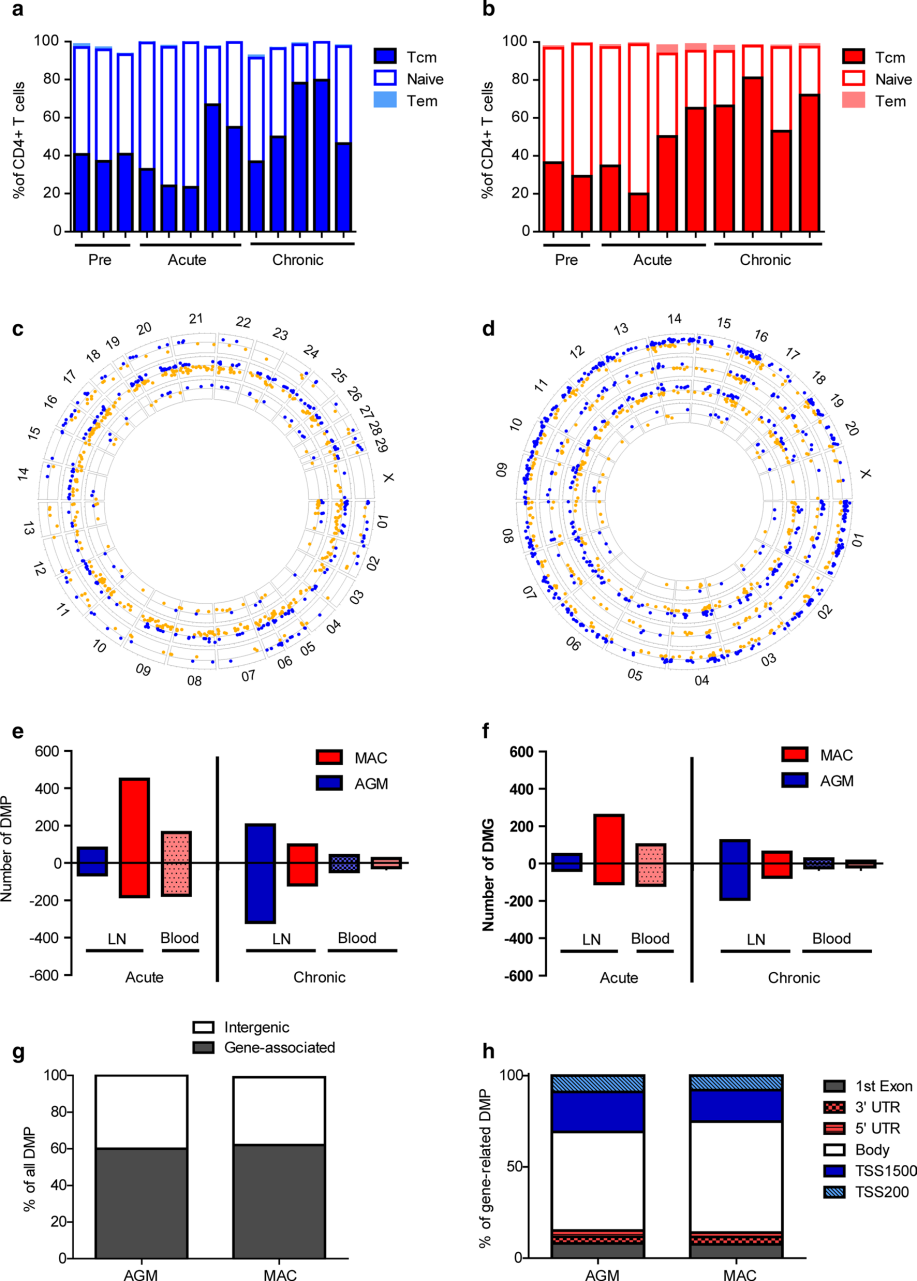
Effect of SIV infection on genome-wide DNA methylation of CD4 + T cells. CD4 + T cell subsets were defined in LN based on CD95 and CD28 expression and divided into central memory (Tcm), effector memory (Tem) and naïve T cells for **a** AGM and **b** MAC. Individual samples are depicted using stacked bar charts. Circular representation of the **c** AGM and **d** MAC genomes, with differentially methylated sites for each of the conditions indicated by dots. Blue and orange dots indicate a hypomethylation and hypermethylation post-infection compared to baseline, respectively. For AGM, the tracks correspond to LN day + 14, LN chronic phase and blood chronic phase from the outside inwards. For MAC, the tracks correspond to LN day + 14, LN chronic phase, blood day + 14 and blood chronic phase from the outside inwards. The numbers of **e** DMP and **f** DMG for AGM (blue) and MAC (red) are depicted. Bars depicting LN are filled with dark red and blue, while bars depicting blood are dotted and filled with light red and blue. Hypomethylated sites are shown as negative numbers and hypermethylated as positive numbers. **g** The percentage of gene-associated and intergenic DMP is shown for AGM and MAC. **h** The distribution of DMP among gene regions is shown. *LN* lymph node, *DMP* differentially methylated probes, *DMG* differentially methylated genes, *TSS* transcription start site, *UTR* untranslated region, *AGM* African green monkeys (*sabaeus*), *MAC* Chinese rhesus macaque. The analyses included for LN and blood, respectively, 5 and 7 MAC at baseline, 4 and 3 MAC in acute infection and 13 and 10 MAC in chronic infection, as well as AGM 5 and 10 AGM at baseline, 4 and 0 in acute infection, and 8 and 9 in chronic infection

Five AGM and five Chinese rhesus MAC were infected with SIVagm._sab92018_ and SIVmac_251_, respectively, and followed longitudinally (Additional file 2: Table 1). The viremia levels were similar in all animals except in one MAC. Already before SIV infection, this animal did not express CD28 on its peripheral CD4 + T cells (Additional file 1^1^ Figure 1A). CD28 is important for immune activation through the T cell receptor [30]. This animal did not control post-acute viremia, did not seroconvert or display T cell activation in blood or lymph nodes and quickly lost CD4 + T cells in the blood (Additional file 1: Figure 1B–G). We therefore excluded this animal from the epigenetic study.

For all other animals, from both species, the infection profile was typical [25, 31, 32]. AGM and MAC showed a median viremia peak at day 9–14 post-infection (p.i.) of 8.6 × 10^7^ and 3.7 × 10^6^ RNA copies/mL blood, followed by a reduction in viremia in the chronic phase to 4.7 × 10^4^ and 2.5 × 10^4^ RNA copies/mL blood, respectively (Additional file 1: Figure 2A). The levels of viral DNA in LN were higher in AGM CD4 + T cells than in MAC CD4 + T cells in the acute phase (Additional file 1: Figure 2B). At 65 days p.i., the DNA viral load had decreased 38-fold in AGM LN compared to day 14 p.i., which corresponds to the reported viral control in LN of AGM [25, 27, 33, 34]. In contrast, MAC displayed only a 4.4-fold decrease compared to day 14 post-infection. MAC partially recovered CD4 + T cell counts (median fold-change of 0.90 at day 62 p.i. compared to baseline) after the acute phase, while AGM had a complete CD4 + T cell recovery (Additional file 1: Figure 2C). AGM showed a lower level of T cell activation in blood and LN than MAC, as expected (Additional file 1: Figure 2D–F). SIV infection led to similar increased CD4 + central memory T cells in lymph nodes in both species, which became in some animals already apparent during acute phase, but was more pronounced during the chronic phase (Fig. 1a, b).

**Fig. 2:**
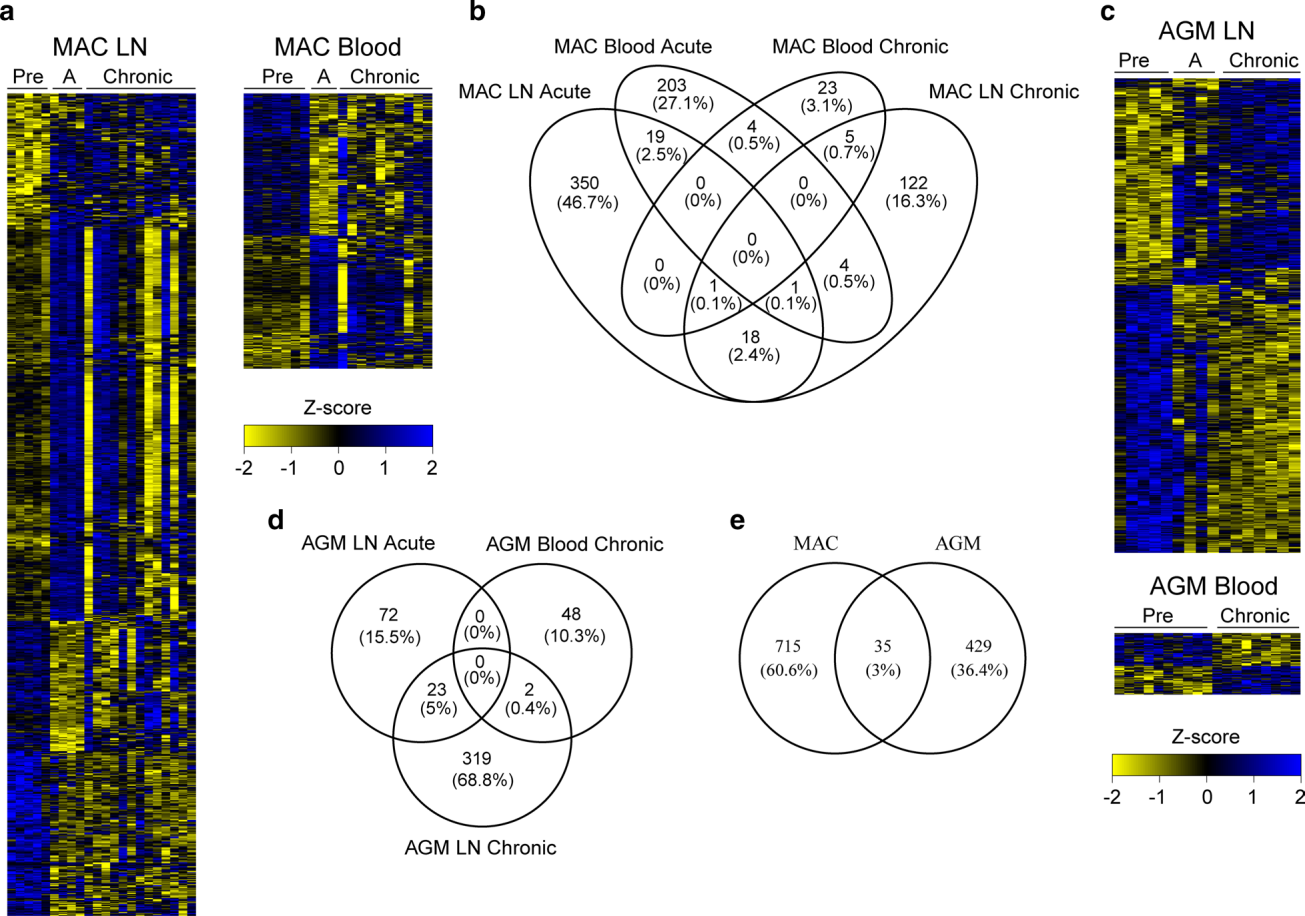
Time and tissue-specific changes in DNA methylation of CD4 + T cells following SIV infection. **a** Heatmaps show methylation levels for each of the differentially methylated probes (DMP) in the rows and individual animals in the columns for MAC LN and blood. Blue indicates highly methylated and yellow indicates low levels of methylation after *z* scale normalization per probe. *LN* lymph node. **a** Acute phase (day14). The height of each of the heatmaps is proportional to the number of differentially methylated probes. **b** Venn diagram showing the number (and percentages in parentheses) of overlapping DMG in the different conditions for MAC. **c** Heatmaps showing methylation levels for AGM as described for MAC above. **d** Venn diagram showing the overlap in DMG for AGM as described for MAC above. **e** Venn diagram showing the overlap in DMG found in both species. *AGM* African green monkeys (sabaeus), *MAC* Chinese rhesus macaque. The number of animals included is the same as in Fig. 1

In summary, the MAC and AGM included in the epigenetic study showed virologic and immunologic parameters similar to those previously reported [25, 31, 32].

### Effect of SIV infection on DNA methylation patterns in CD4 + T cells in vivo

Genome-wide changes in DNA methylation in CD4 + T cells of LN and blood were analyzed longitudinally in the 5 AGM and 4 MAC described above. DNA methylation was measured at three timepoints: before infection, during acute (day 14 p.i.) and early chronic infection (day 62–65 p.i.). In addition, we analyzed pre-infection and chronic infection samples (after day 200 p.i.) from another fourteen AGM and twelve rhesus MAC previously described (Additional file 1: Figure 3 and Additional file 2: Table 1) [32, 35–37].

**Fig. 3:**
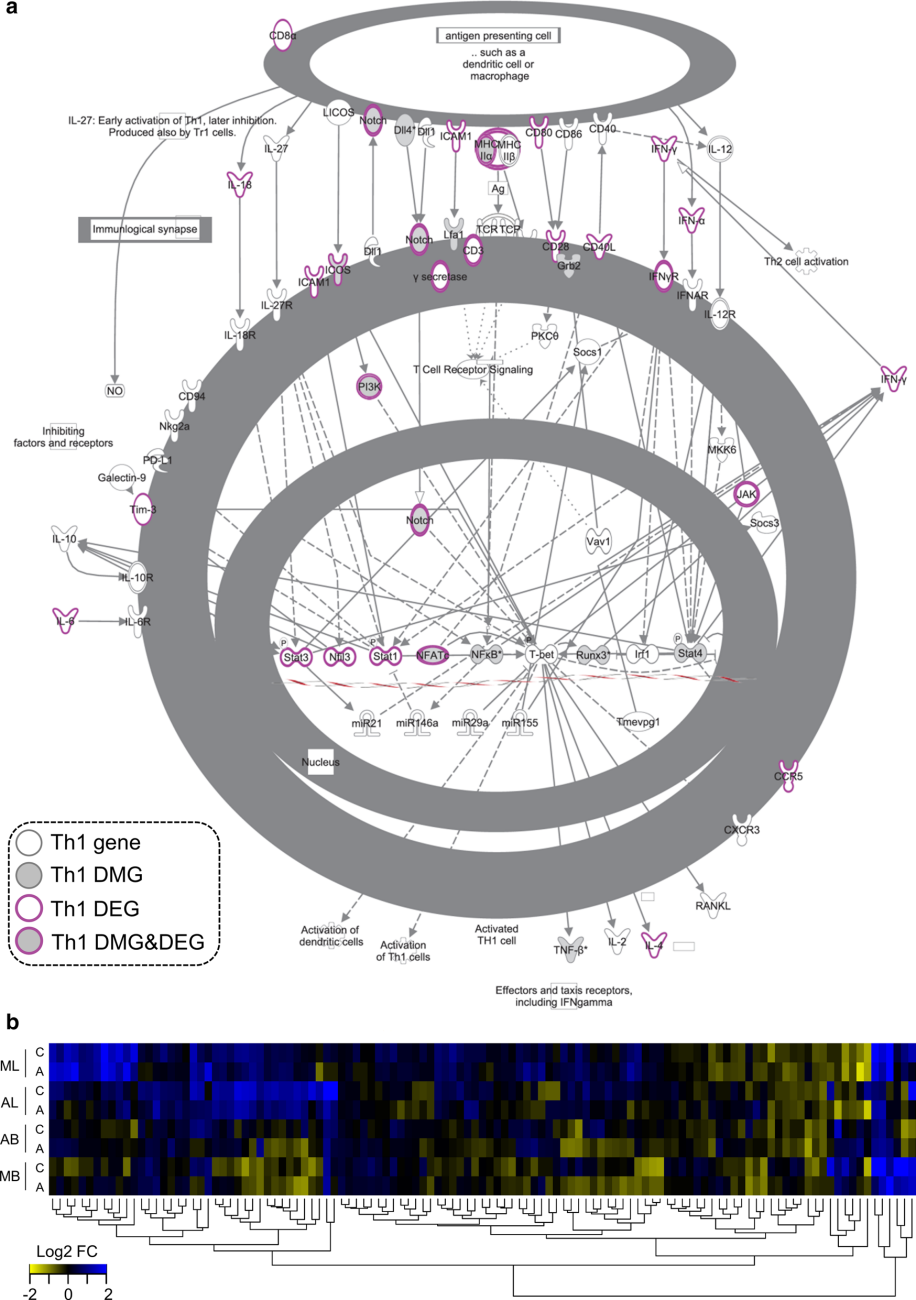
Effect of SIV infection on the Th1 pathway using ingenuity pathway analysis. **a** The canonical Th1 pathway is depicted, with genes within this pathway indicated as symbols and lines indicating regulation. Genes of the canonical Th1 pathway that were differentially methylated (DMG) in MAC are shown in grey; other genes are shown in white. Genes that were differentially expressed (DEG) have a purple border. Some genes were both differentially methylated and differentially expressed following infection (DMG and DEG) and are shown in grey with a purple border. **b** Heatmaps of gene expression of Th1-related genes during SIV infection. Columns correspond to conditions (species, tissue, phase of infection) and rows to individual genes. The mean log_2_ fold-changes to baseline are indicated, with blue corresponding to increased expression and yellow to decreased expression. Six AGM (sabaeus) and six Chinese rhesus macaques were analyzed for gene expression during acute (day + 14) and chronic infection (day + 65) from blood, and six and five animals per species were analyzed for acute and chronic infection, respectively, in LN. MB = MAC blood, AB = AGM blood, ML = MAC lymph node, AL = AGM lymph node, A = acute, C = chronic

Using the Infinium HumanMethylation450K BeadChip (Illumina) by restricting the analysis to a subset of probes that target CpGs at a unique location on the MAC and AGM genomes, we were able to study methylation at 157,164 and 154,108 CpG sites for AGM and MAC, respectively [38]. As expected, genome-wide methylation pattern showed a typical bimodal distribution (Additional file 1: Figure 4A) and a strong correlation was observed when all probes were compared between MAC and AGM (Additional file 1: Figure 4B). The overall distribution within the genome of the differentially methylated sites was therefore similar between AGM and MAC.

**Fig. 4:**
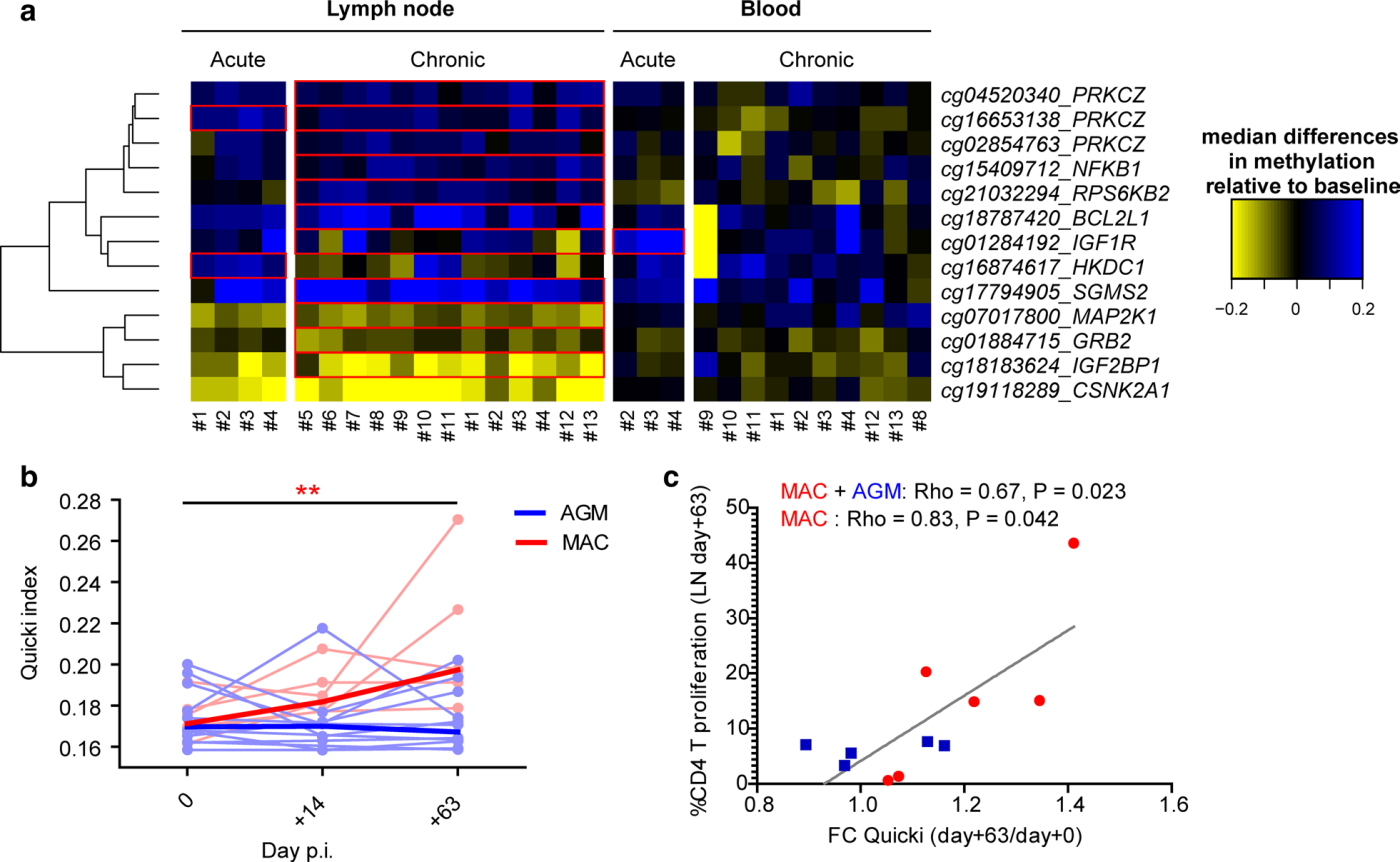
Analysis of the changes in DNA methylation of metabolic genes and of insulin sensitivity before and after SIVmac infection in macaques. **a** Heatmap showing median changes in methylation levels of insulin-signaling and insulin growth factor 1/2-signaling genes during SIVmac infection. Columns correspond to individual animals (rhesus macaques) and rows to probes. Blue indicates increased methylation and yellow decreased methylation compared to baseline, defined as the median of expressions of the pre-infection samples (blood: *n* = 5 pre-infection, 3 acute infection, 10 chronic infection; lymph node: *n* = 7 pre-infection, 4 acute infection, 13 chronic infection). Probes with a statistically significant change as compared to baseline are marked by a red box. **b** Insulin sensitivity is shown for MAC (red, *n* = 6) and AGM (blue, *n* = 11). Individual animals are shown in light color and median in dark color. **p < 0.01 compared to baseline using Friedman test and Dunn’s multiple comparison. **c** Correlation between change in insulin sensitivity following SIV infection and CD4 + T cell proliferation in LN is shown. Individual animals (MAC, *n* = 6 in red and AGM, *n* = 5 in blue) and linear regression results are depicted (for all animals combined). Pearson rho and *p* value are shown for either MAC alone or for MAC and AGM combined

For AGM LN CD4 + T cells, we identified 143 differentially methylated probes (DMP, 79 hypermethylated and 64 hypomethylated) during the acute phase and 521 DMP (203 hypermethylated and 318 hypomethylated) during the chronic phase of SIV infection when compared to baseline (Fig. 1c, e). In MAC LN CD4 + T cells, 630 DMP (449 hypermethylated and 181 hypomethylated) were identified in the acute phase, while 214 DMP (96 hypermethylated and 118 hypomethylated) were identified in the chronic phase (Fig. 1d, e). In blood CD4 + T cells from AGM, 86 DMP were identified in the chronic phase (40 hypermethylated and 46 hypomethylated). In MAC, we identified 336 DMP in blood in the acute phase (163 hypermethylated and 173 hypomethylated) and 49 in the chronic phase (24 hypermethylated and 25 hypomethylated).

The majority of the DMP were associated with genes (DMG): 62% for MAC and 60% for AGM (Fig. 1f, g). Gene-associated DMP mapped most frequently to gene promoters and gene bodies: 30.9% and 25.2% mapped to promoters for AGM and MAC, respectively, while 54% and 60.8% of DMP mapped to gene bodies (Fig. 1h). The remainder of gene-associated DMP mapped to the first exon or 5′ UTR or 3′ UTR (Fig. 1h). The complete list of DMP and associated genes is shown in Additional file 3: Table 2.

Thus, this analysis indicates that SIV infection modifies DNA methylation pattern in a substantial number of genes within CD4 + cells. MAC and AGM both showed more changes in LN than in blood, MAC mostly during acute infection and AGM predominantly during chronic infection.

### Changes in DNA methylation dependent on tissue and stage of SIV infection

We next compared the tissue-dependent changes in DNA methylation for AGM and MAC (Fig. 2a–d). Only few genes were similarly affected in blood and LN CD4 + T cells, suggesting that changes were tissue-specific. For MAC, 25 out of 750 (3.3%) DMG were found in both LN and blood. These included genes encoding cytokines and transcription factors, such as *LTA* (lymphotoxin alpha), *FOXP1* (forkhead box protein 1) and *TRIM26* (tripartite motif containing 26) (Additional file 3: Table 2). In AGM, only 2 out of 464 DMG were found in both tissues (0.4%): *CDHR5* (cadherin-related family member 5) and *LRRC57* (leucine-rich repeat containing 57).

We then analyzed time-dependent DNA methylation changes in blood and LN. The DNA methylation profiles in acute and chronic infection were closer to each other than to the profiles before infection in both blood and LN and for both AGM and MAC (Fig. 2). DNA methylation profiles still considerably differed between acute phase and chronic infection in both species (Fig. 2a, c). In MAC LN, 20 out of 520 DMG were found in common between acute and chronic infection (3.8%). These DMG included genes encoding proteins with known immunological roles such as KIT ligand (*KITLG*), Runt-related transcription factor 3 (*RUNX3*) and Protein kinase C zeta (*PRKCZ*). In MAC blood, 4 out of 260 DMG were found in common between acute and chronic infection (1.5%). These genes included the immune genes encoding major histocompatibility complex, class II, DM alpha (*HLA-DMA*) and interleukin-5 receptor subunit alpha (*IL5RA*). Similarly, in AGM LN, 23 out of 414 (5.6%) of DMG were common between both timepoints. Four out of these were small nucleolar RNAs (snoRNA): *SNORA1*, *SNORA32*, *SNORA8* and *snoZ40*. In total, 698 (93.0%) and 439 (94.6%) of all DMG were unique to a timepoint and tissue for MAC and AGM, respectively (Fig. 2b, d).

Thirty-five DMG (3%) were identified common in both species (Fig. 2e). Genes that were common between the two species included *TRIM26* and growth factor receptor-bound protein 2 (*GRB2*).

Taken together, the changes in DNA methylation detected following SIV infection were most often different between blood and LN, dynamic over time and species-specific. This emphasizes the need of analyzing tissues during infection. The analysis revealed differential methylation in genes, such as *FOXP1*, *TRIM26*, *LTA*, *RUNX3* and *HLA* upon SIV infection.

### DNA methylation changes in pathways related to Th1 in SIVmac infection and to tissue integrity in SIVagm infection

We investigated among the DMG whose pathways were specifically enriched in MAC or AGM. In the first approach, we used all genes that were identified as differentially methylated following SIV infection, combining both tissues and timepoints. The most significantly increased pathways in MAC were that of colorectal cancer metastasis signaling (*p* = 10^–4.4^), Th1 pathway (*p* = 10^–3.2^), Wnt/B-catenin signaling (*p* = 10^–3.2^) and retinoic acid receptor (RAR) activation (*p* = 10^2.7^) (Table 1). These pathways included many genes related to cell proliferation and inflammation, such as the E2F transcription factor 4 (*E2F4)*, mitogen-activated protein kinase *MAP2K1*, guanine nucleotide binding protein (G Protein), alpha stimulating (*GNAS*), Nuclear Factor Kappa B Subunit 1 (*NFKB1*), BCL2 Like 1 (*BCL2L1*) and tumor necrosis factor alpha (*TNF*) and also to T cell differentiation. A total of 13 genes of the Th1 and T_FH_ pathways were differentially methylated, including signal transducer and activator of transcription 4 (*STAT4*), *NOTCH4*, inducible T cell costimulator (*ICOS*), nuclear factor of activated T cells (*NFATC2*) and major histocompatibility complex class II DO alpha (*HLA-DOA*) (Fig. 3).

**Table 1:** Canonical pathways among total rhesus macaque DMG

Ingenuity canonical pathways	− log(*p* value)	Genes
Colorectal cancer metastasis signaling	4.42	*SRC, LRP5, MMP20, E2F4, GNAS, WNT3, GRB2, AXIN1, PIK3R1, ADCY6, NFKB1, MAPK12, BCL2L1, WNT3A, WNT10A, RND3, ADCY5, MMP26, DCC, TNF, MAP2K1, TCF7L2*
Th1 pathway	3.23	*STAT4, ITGB2, RUNX3, NOTCH4, HLA-DOA, HLA-DMA, GRB2, LTA, PIK3R1, ICOS, NFATC2, NFKB1, DLL4*
Wnt/β-catenin signaling	3.17	*UBD, SRC, SOX7, LRP5, CSNK2A1, WNT3A, WNT10A, WNT3, DKK3, AXIN1, RARA, TLE3, MAP4K1, TCF7L2, SOX5*
RAR activation	2.65	*SRC, CSNK2A1, CYP26A1, SDR16C5, PIK3R1, ADCY6, MAPK12, NFKB1, PRKCZ, PRMT1, ALDH1A3, ADCY5, RARA, CRABP2, MAP2K1*
Toll-like receptor signaling	2.44	*SIGIRR, UBD, TICAM2, TNFAIP3, MAPK12, NFKB1, TNF, IRAK2*
Acute myeloid leukemia signaling	2.40	*RUNX1, KITLG, GRB2, PIK3R1, RARA, RPS6KB2, NFKB1, MAP2K1, TCF7L2*
Melanocyte development and pigmentation signaling	2.25	*KITLG, SRC, GNAS, GRB2, ADCY5, PIK3R1, RPS6KB2, ADCY6, MAP2K1*
Role of NFAT in cardiac hypertrophy	2.21	*SRC, LIF, HDAC4, GNAS, GRB2, PIK3R1, ADCY6, MAPK12, PRKCZ, ADCY5, IGF1R, CAMK1G, SLC8A1, MAP2K1*
GABA receptor signaling	2.12	*UBD, SLC6A11, GNAS, AP1G2, ADCY5, ADCY6, GABBR1*
Neuregulin signaling	2.01	*SRC, NRG2, GRB2, PIK3R1, DCN, RPS6KB2, MAP2K1, PRKCZ*

Top 10 pathways sorted by statistical significance are depicted

The most significantly increased pathways altered by SIV infection in AGM corresponded for instance to neuregulin signaling (*p* = 10^–3.3^), nitric oxide signaling (*p* = 10^–3.3^) and caveolar-mediated endocytosis signaling (*p* = 10^–3.1^). These pathways included genes related to signaling and cell proliferation (protein kinase C, epidermal growth factor receptor), immunosuppression (arginase), protein folding (heat shock protein 90), endocytosis and gap junctions (Table 2).

**Table 2:** Canonical pathways among total AGM DMG

Ingenuity canonical pathways	− log(*p* value)	Genes
Neuregulin signaling	3.34	*RPS6KB1, ITGA3, HSP90AB1, GRB2, CRKL, PRKCB, EGFR, PRKCA*
Nitric oxide signaling in the cardiovascular system	3.33	*PDE2A, HSP90AB1, GUCY1A3, GRB2, PRKAR1B, PIK3CD, ARG2, PRKCB, PRKCA*
Caveolar-mediated endocytosis signaling	3.14	*ITGA3, ITGA7, ACTC1, ITGB7, COPG1, EGFR, PRKCA*
Cholecystokinin/gastrin-mediated signaling	2.88	*PTK2, GRB2, CREM, PLCB1, IL1F10, PRKCB, EGFR, PRKCA*
3-phosphoinositide biosynthesis	2.64	*PLPP6, ITPK1, TNS3, PTPRJ, PPP1R7, GRB2, DOT1L, PIK3CD, MTMR7, EGFR, SACM1L*
HER-2 signaling in breast cancer	2.59	*GRB2, PIK3CD, PARD3, ITGB7, PRKCB, EGFR, PRKCA*
Gap junction signaling	2.59	*DBN1, GUCY1A3, GRB2, PRKAR1B, PLCB1, PIK3CD, ACTC1, PRKCB, EGFR, PRKCA*
UVB-induced MAPK signaling	2.58	*RPS6KB1, GRB2, PIK3CD, PRKCB, EGFR, PRKCA*
HGF signaling	2.54	*ETS1, PTK2, ITGA3, GRB2, CRKL, PIK3CD, PRKCB, PRKCA*
Superpathway of inositol phosphate compounds	2.53	*PLPP6, ITPK1, TNS3, PTPRJ, PPP1R7, GRB2, DOT1L, PLCB1, PIK3CD, MTMR7, EGFR, SACM1L*

Top 10 pathways sorted by statistical significance are depicted

Collectively, in SIV-infected MAC, significant alterations were found in cell cycling and Th1 genes, while in SIV-infected AGM the alterations were rather linked to cell growth, immune regulation and tissue integrity.

### SIVmac infection induces DNA methylation changes in metabolic pathways in lymph nodes

Given the deleterious impact of HIV persistence and chronic immune activation on the immune responses and the relevance of the virus–host interactions within LN, we performed an enrichment analysis focusing on the LN from MAC. DMG observed during the chronic phase were compared to baseline (Table 3). Three of the most significantly enriched pathways were related to metabolism, such as phosphatase and tensin homolog (PTEN) signaling, phosphatidylinositol 3-kinase (PI3K)/AKT signaling and insulin-like growth factor 1 (IGF-1) signaling. DMG associated with these pathways included *MAP2K1*, *PRKCZ*, growth factor receptor-bound protein 2 (*GRB2*) and *RPS6KB2* (ribosomal protein S6 kinase B2).

**Table 3:** Canonical pathways among rhesus macaque DMG in LN during chronic phase of the infection

Ingenuity canonical pathways	− log(*p* value)	Genes
PTEN signaling	4.73	*BCL2L1, CSNK2A1, GRB2, RPS6KB2, NFKB1, MAP2K1, PRKCZ*
PI3K/AKT signaling	3.69	*BCL2L1, GRB2, RPS6KB2, NFKB1, MAP2K1, PRKCZ*
Acute myeloid leukemia signaling	3.40	*KITLG, GRB2, RPS6KB2, NFKB1, MAP2K1*
CD27 signaling in lymphocytes	3.37	*BCL2L1, BID, NFKB1, MAP2K1*
IGF-1 signaling	3.07	*CSNK2A1, GRB2, RPS6KB2, MAP2K1, PRKCZ*
NGF signaling	2.90	*GRB2, RPS6KB2, NFKB1, MAP2K1, PRKCZ*
ErbB4 signaling	2.85	*YAP1, GRB2, MAP2K1, PRKCZ*
Role of NANOG in mammalian embryonic stem cell pluripotency	2.83	*WNT3A, RIF1, WNT3, GRB2, MAP2K1*
IL-15 signaling	2.72	*BCL2L1, GRB2, NFKB1, MAP2K1*
Erythropoietin signaling	2.66	*GRB2, NFKB1, MAP2K1, PRKCZ*

Top 10 pathways sorted by statistical significance are depicted

We further investigated the observed changes in DNA methylation in MAC regarding insulin-signaling- and IGH-signaling-related genes by looking at the 13 corresponding probes within these pathways (Fig. 4a). The majority of differential methylation was observed in LN (10 genes in LN and 1 gene in blood). Three insulin-signaling- and IGH-signaling-related genes were differentially methylated during the acute phase of SIVmac infection and 9 during chronic infection. This enrichment during the chronic phase was even more notable given that the number of total genome-wide differentially methylated probes in LN was higher during the acute than chronic SIVmac infection (Fig. 1b, c).

To test whether the changes in DNA methylation were associated with functional alterations in metabolism, we investigated insulin sensitivity before and during SIVagm and SIVmac infection. Levels of glucose and insulin were measured in both AGM and MAC, and insulin sensitivity was calculated using the Quicki index (Fig. 4b) [39]. Only MAC displayed changes in insulin sensitivity in chronic infection (*p* < 0.01), while AGM did not. The magnitude of this increase in insulin sensitivity correlated with levels of CD4 + T cell proliferation in LN (Fig. 4c).

To better understand the exquisite control of viral load and inflammation in AGM LN during chronic infection, we performed pathway enrichment analysis similar as described above. In contrast to MAC, we did not detect any significantly enriched pathways for AGM LN.

Thus, these findings link changes detected in DNA methylation together with functional changes in metabolism and immune activation during SIV infection. They highlight the rapid and profound metabolic changes in acute SIVmac infection in LN, contrasting with the paucity in LN during SIVagm infection.

### Relation between the detected changes in DNA methylation and gene expressions

**Fig. 5:**
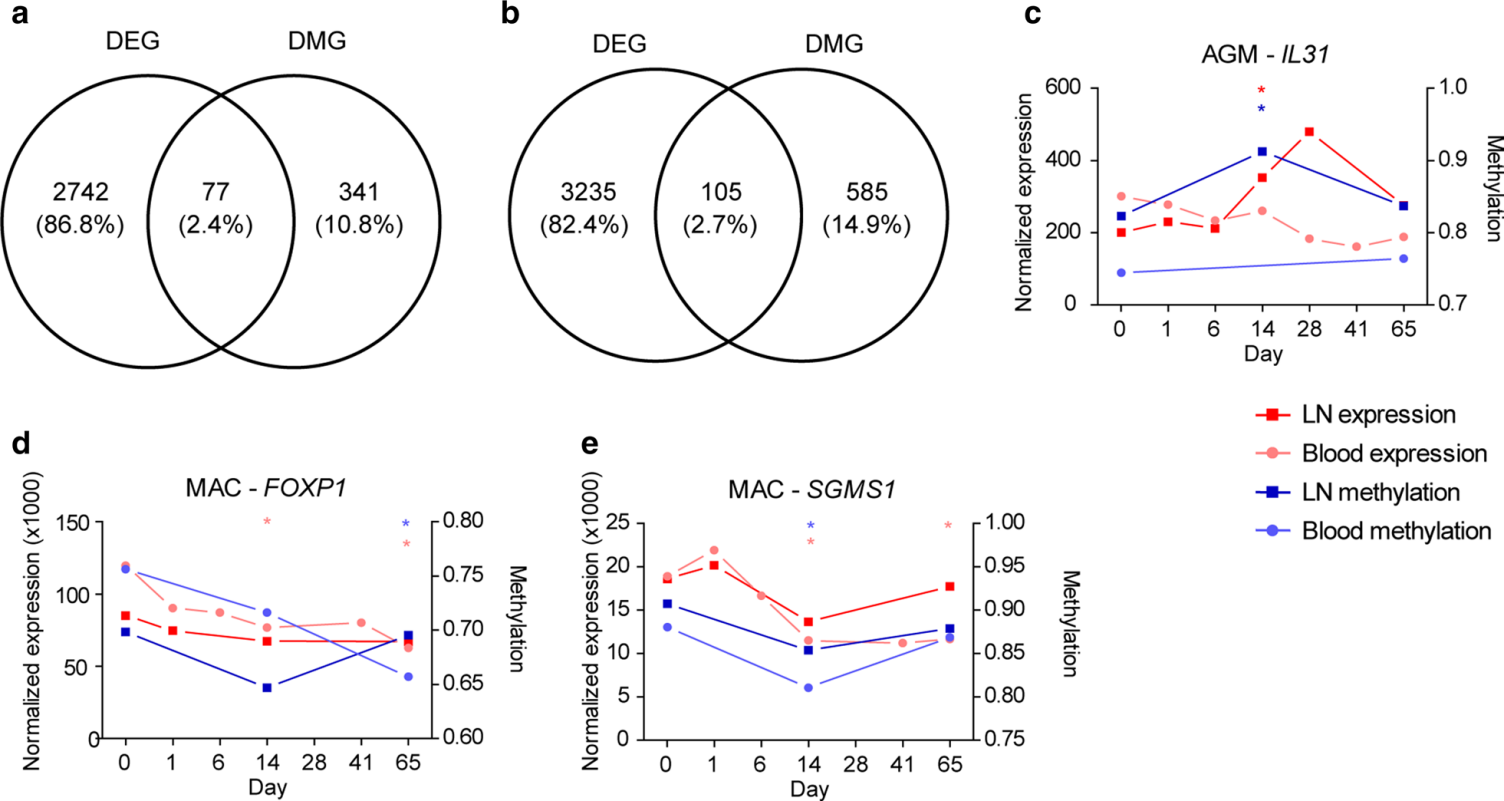
Integration of changes in DNA methylation and changes in gene expression levels during SIV infection. **a** AGM and **b** MAC Venn diagrams showing the number of differentially methylated genes (DMG) and differentially expressed genes (DEG). Percentages within parentheses. Only genes that were present in both microarray types were included. Example plots showing methylation levels and expression levels for **c** AGM *IL31* (cg09750599) (*n* = 4–10), **d** MAC *FOXP1* (cg05384123) and **e** MAC *SGMS1* (cg10631515) (*n* = 3–13). Methylation is indicated with blue (right axis) and expression with red (left axis). Mean levels are shown for lymph node (LN, dark) and blood (light). Baseline levels are denoted as day 0. *Indicates significant difference compared to pre-infection as described in the methods, with the color corresponding to its condition

To further test the relevance of the changes in DNA methylation that we have observed during SIV infection, we analyzed if the genes that were differentially methylated also display differential expression levels. For doing so, we used transcriptome data of CD4 + T cells. These data were from a different cohort of six AGM and MAC that we previously reported [32]. The twelve animals were of the same species, infected with the same virus, same infectious dose, same infection route, in a similar environment, and CD4 + T cells from blood and LN were isolated in exactly the same way and at the same timepoints as the AGM and MAC in the current study. For AGM, a total of 2819 differentially expressed genes (DEG, adjusted *p* value < 0.05 and fold change > 1.5) were identified, combining all sites and infection stages. Of all AGM DMG, 77 DMG (18.4%) were in differentially expressed genes (Fig. 5a). Among these genes were *IL-21*, *IL-31* and lymphocyte activating 3 (*LAG3*). For MAC, a total of 3340 differentially expressed genes were identified in total, and 105 DMG (15.2%) were in differentially expressed genes (Fig. 5b), including interleukin-1 receptor associated kinase 2 (*IRAK2*), protein inhibitor of activated STAT1 (*PIAS1*), *PRKCZ*, *FOXP1*, TNF alpha-induced protein 3 (*TNFAIP3* or A20) and Wnt family member 3 (*WNT3*). The complete list of genes that were both differentially methylated and expressed is shown in Additional file 4: Table 3 and the methylation and expression profiles for three genes are exemplified in Fig. 5c–e.

To further test the relevance of the detected profiles, we also analyzed if there was a concordance between DNA methylation and gene expression changes in the Th1 pathway. Of the 71 genes included in the Th1 pathway (ingenuity pathway analysis), 33 genes were differentially expressed (DEG) following SIVmac infection (Fig. 3b). Three of these genes were both differentially methylated and differentially expressed: *ICOS*, *NFATC2*, *HLA-DOA.*

Overall, we observed an overlap between DNA methylation and gene expression in as much as 15–18% of the genes. The analysis reveals potential novel targets of DNA methylation during SIV infection, such as *Runx3*, *IL21* and *IL31*.

### Validation of DNA methylation changes in an independent animal cohort

**Fig. 6:**
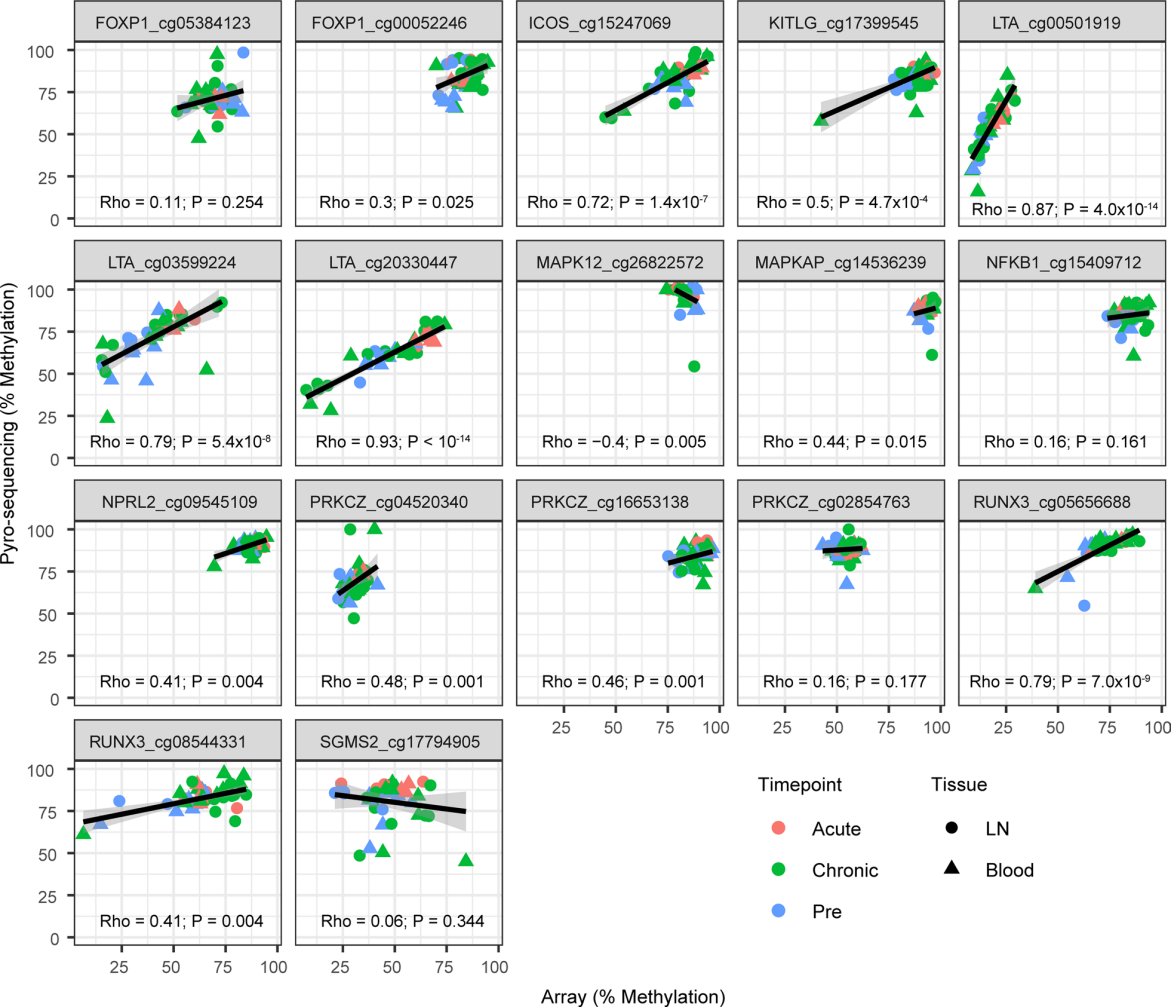
Comparison of methylation levels by bisulfite pyro-sequencing and microarrays. Symbols denote individual animals. Forty-two samples from Chinese rhesus macaques were analyzed. Cells from LN and blood are depicted as circles and triangles, respectively. Symbol color corresponds to timepoint with pre-infection (blue), acute infection (red) and chronic infection (green) shown. Linear regression lines are shown, and spearman rho and *p* value s are indicated per probe

We furthermore aimed to validate the identified differentially methylated sites using an orthogonal technology. Moreover, we investigated whether the changes in DNA methylation we observed were distinct between viremic and non-viremic SIVmac infections. Therefore, we developed bisulfite pyrosequencing assays to investigate probes that were differentially methylated during SIVmac infection. We selected 17 differentially methylated probes in 11 genes that were either: differentially methylated in more than one tissue or timepoint; and/or were also differentially expressed following infection; and/or possess a known important immunological or metabolic function. These selected genes were *ICOS* (affected by DNA methylation in blood during acute infection, DEG), *FOXP1* (LN and blood chronic infection, DEG), *LTA* (LN and blood chronic infection), *MAPK12* (blood acute and chronic infection), MAPK-associated protein 1 (*MAPKAP1*, blood chronic infection), nitrogen permease regulator 2-like protein (*NPRL2) and RUNX3* (LN acute and chronic), *PRKCZ* (LN acute and chronic, DEG), *NFKB1* and sphingomyelin synthase 2 (*SGMS2*, LN chronic infection). Levels of DNA methylation measured by pyrosequencing significantly correlated with those observed by microarray for 12 out of 17 probes when re-analyzing the same samples (Fig. 6).

**Fig. 7:**
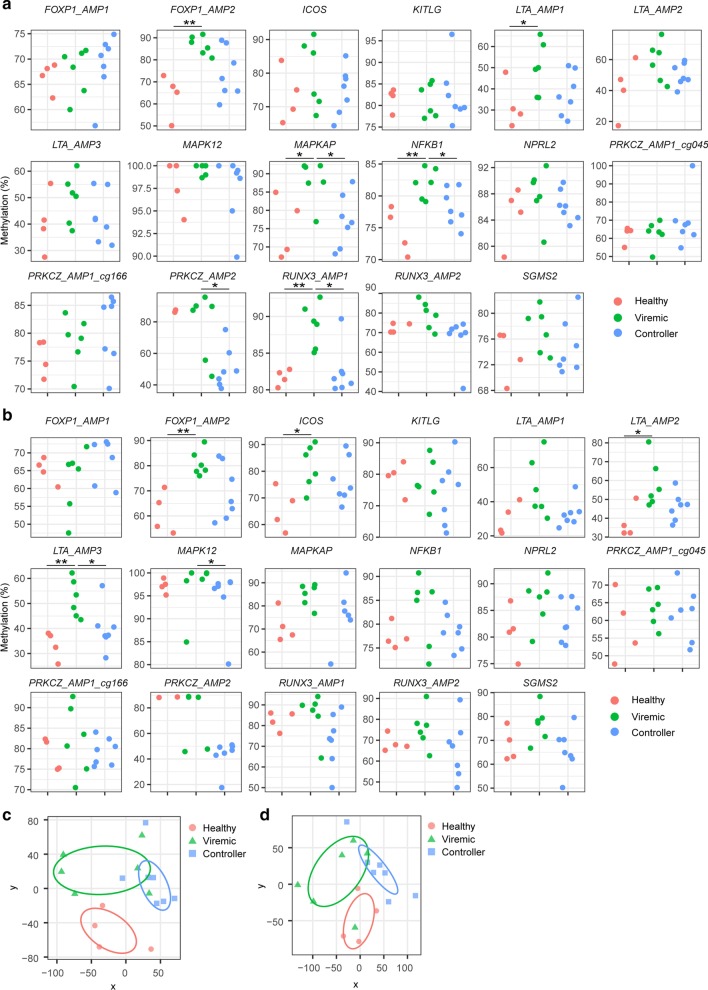
Validation of methylation changes in an independent cohort of 17 Indian rhesus macaques. Comparison of DNA methylation levels at the 17 selected probe sites in the validation cohort by sequencing CD4 + T cells from **a** LN and **b** blood. *p < 0.05, Mann–Whitney, **p < 0.01, Mann–Whitney. **c** LN and **d** blood multidimensional scaling showing clustering of validation animals for all CpG included in the 16 bisulfite pyrosequencing assays. Symbols represent individual animals that are uninfected (red circles, *n* = 4), infected and viremic (green triangles, *n* = 6) or SIV controllers (blue squares, *n* = 7). Ellipses depict 50% confidence intervals. Kruskal stress is 0.14 and 0.13 for LN and blood, respectively

DNA methylation was then measured in CD4 + T cells isolated from LN and blood from an unrelated colony of 17 Indian rhesus macaques (Additional file 1: Figure 3). Animals were either uninfected with SIV (*n* = 4), or chronically infected with SIVmac239 or SIVmac251 (*n* = 13). Seven of the 13 SIV-infected animals controlled viremia below the limit of detection (37.5 copies/mL) and were defined as SIV controllers. The other six SIV-infected animals had a median viral load of 2.2 × 10^5^ (range 5 × 10^4^–3.6 × 10^6^). *FOXP1*, *LTA*, *MAPKAP*, *NFKB1* and *RUNX3* were differentially methylated in LN between uninfected and infected viremic animals using pyrosequencing analysis, confirming our microarray results (Fig. 7a). *MAPKAP*, *NFKB1, PRKCZ* and *RUNX3* methylation levels were significantly increased in viremic compared to SIV controller animals, with no significant differences between uninfected and SIV controller animals for any probe site. In blood CD4 + T cells, methylation levels in *FOXP1, ICOS* and *LTA* were significantly increased in viremic animals versus uninfected animals (Fig. 7b). Viremic animals also showed increased methylation in *LTA* and *MAPK12* compared to SIV controller animals, with again no significant differences between uninfected and SIV controller animals for any probe site. Multidimensional scaling analysis on all methylation sites covered within the probes demonstrated that SIV-infected animals clustered separately from uninfected animals with little overlap between viremic and SIV controller animals (Fig. 7c, d).

Taken together, the changes in DNA methylation identified using microarrays were confirmed using bisulfite sequencing in an independent animal cohort. This was the case for example for *RUNX3*, *FOXP1*, *LTA* and *NFKB1*. Moreover, the analysis also showed that in contrast to viremic macaques, the selected DNA methylation sites in blood and LN CD4 + T cells from SIV controller animals were not different from those of uninfected animals.

## Discussion

**Fig. 8:**
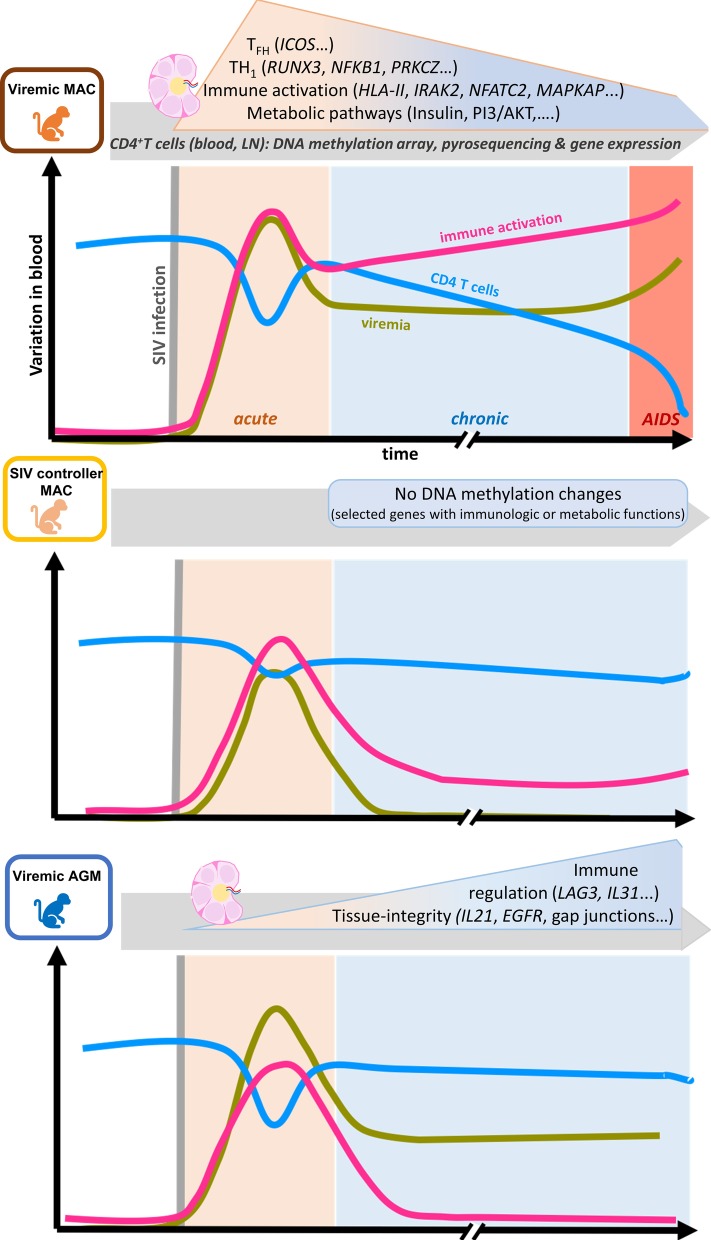
Model depicting DNA print changes in pathogenic and nonpathogenic SIV infection. *MAC* macaque, *AGM* African green monkeys

In this study, we measured genome-wide DNA methylation changes following SIV infection in CD4 + T cells of two compartments (blood and LN) longitudinally at three timepoints (before, acute and chronic infection). Genes of interest were confirmed as being differentially methylated using both an independent cohort of MAC and an ortholog method (bisulfite pyrosequencing). Functional relevance of the identified changes in DNA methylation was further confirmed in vivo by gene expression analysis and blood insulin sensitivity analyses. In viremic MAC, differentially methylated genes were found to be enriched in Th1 differentiation and metabolic pathways, including IGF-1 signaling (Fig. 8). The analyses further showed that AGM display DNA methylation patterns different from MAC upon SIV infection with preferential changes in genes associated with immune regulation and tissue growth or repair. SIV controllers displayed a distinct profile that was similar to the non-infected animals. DNA methylation changes were identified already in primary infection and were more abundant in lymph node than blood CD4 + T cells.

One potential limitation of this study is that human microarrays were used to measure DNA methylation on non-human primate genomes. However, alignment and stringent quality control of the included probes to the AGM and MAC genomes revealed that still 162,053 and 158,970 probes could be confidently used covering 16,295 and 16,611 genes, respectively [38]. Of those, 157,164 and 154,108 probes were detected with high confidence in the current study. Moreover, we confirmed differential DNA methylation in genes such as *FOXP1*, *LTA*, *MAPKAP*, *NFKB1*, *PRKCZ* and *RUNX3* by bisulfite pyrosequencing. In addition, some genes for which methylation patterns were particularly stable over time after infection (HLA, detected both in acute and in chronic in SIV-infected macaques) correspond to those also reported as most often differentially methylated in HIV-infected individuals in a study of whole blood during chronic infection [17]. Similarly, CpG associated with *ICOS, FOXP1*, HLA-II, *NOTCH4* and *TRIM* were found differentially methylated in whole blood from HIV-infected children under anti-retroviral therapy compared to non-infected children [18]. The fact that our findings in part replicate earlier work and that we identified similar genes in independent animals gives further credence to this study and earlier reports.

Another limitation to our study was that DNA methylation and gene expression were not performed on the same samples, but on two separate cohorts. This prevented us from fully integrating changes in DNA methylation and gene expression on an individual animal level, and we therefore were restricted to comparing gene lists. Around 15–18% of the genes that were differentially methylated after SIV infection were also differentially expressed. The range was the same between AGM and MAC and also similar to those described in previous reports [40–42]. Depending on location, DNA methylation can activate or repress gene expression or lead to alternative splicing [43]. In MAC, DMG were enriched for Th1 differentiation, which was mirrored by changes in gene expression in the same pathway. In particular, three genes (*ICOS*, *NFATC2* and *HLA-DOA)* were both differentially methylated and expressed. Moreover, several key transcription factors in this pathway were differentially methylated: *STAT4*, *RUNX3* and *NFKB1.* This is in line with previous reports in the literature indicating a stronger Th1 response in MAC compared to natural hosts [23, 26]. These data also agree with the notion that during chronic HIV/SIVmac infection, increased levels of follicular T helper cells with Th1 characteristics (T_FH_1) are found, which could potentially contribute to driving the chronic interferon signature observed during pathogenic infections [44]. The observation that a gene such as *RUNX3*, which is important for Th1 function [45], is already differentially methylated during acute infection might be an indication that this mechanism could enforce cell fates and/or be actively involved in maintaining Th cell subset disbalance during HIV/SIV infection.

We cannot exclude that some of the alterations in DNA methylation we observed are due to dynamic changes over time in cell composition. However, DNA methylation changes were detected already during the acute phase of infection (day 14 p.i.), before we observed the largest differences in memory phenotype. Moreover, several of the genes observed as differentially methylated in our study have previously been found differentially methylated in inflammatory disorders and autoimmune diseases characterized by an interferon DNA methylation signature including systemic lupus erythematosus (*LTA*, *RUNX3*, *NFATC2*, *PRKCZ)* [46–48], Behçet’s disease (*STAT4*, *RUNX3*, *PRKCZ*) [49], multiple sclerosis (*RUNX3*, *HLA-DOA*, *PRKCZ*) [50, 51], primary Sjøgren’s syndrome (*LTA*, *RUNX3*, *NFATC2*, *HLA-DOA*, *NFKB1*, *PRKCZ*) [52–54] and rheumatoid arthritis (*NFATC2*, *ICOS*, *NFKB1*, *PRKCZ*) [55–57]. Furthermore, the differentially methylated genes *NFATC2*, *STAT4*, *HLA-DOA*, *FOXP1*, *ICOS*, *LTA*, *MAPK12*, *NFKB1, PRKCZ* and *RUNX3* have all been identified as interferon regulated genes [58]. This indicates the existence of common activated pathways between viral-induced inflammation and non-infectious inflammatory disorders.

We show that SIV infection in MAC led to alterations in DNA methylation in metabolic pathways, including IGF-1 signaling. Previously, insulin signaling and type 2 diabetes have been found to be associated with changes in DNA methylation [59, 60]. A study in which a HIV-infected and uninfected twin were studied identified a differential DNA methylation in the gene *IGFBP6*, which inhibits IGF-2 signaling [19, 61]. Insulin signaling regulates the capacity of cells to take up glucose, which is the energy source for activated T cells that switch to aerobic glycolysis [62]. Indeed, insulin receptor signaling in T cells is associated with increased effector function [63]. Our study suggests an increase in insulin sensitivity during the very early chronic phase of SIVmac infection (day 62 p.i.). This contrasts with other studies showing an insulin resistance in HIV infection. However, increases in insulin sensitivity have previously been observed in HIV infection [64]. Furthermore, we studied insulin sensitivity only in the early phase of chronic SIV infection (day 63 p.i.). The alteration in CD4 + T cell DNA methylation in metabolic pathways was especially pronounced in LN. The lymphatic system plays a role in whole-body metabolism via lipid transport and is thought to be involved in the pathogenesis of obesity and type 2 diabetes [65]. In a more general perspective, our study indicates that some of the metabolic changes induced by SIV infection are epigenetically fixed already soon after infection. This might in part explain why ART generally does not revert all HIV-induced abnormalities in the CD4 + T cells or why it might take many years to fully revert.

Our study uncovered that DNA methylation patterns in CD4 + T cells of SIV controller MAC were more similar to uninfected than to viremic animals. Only a few studies have analyzed the transcriptional profile of the whole CD4 + T cell compartment in HIV controllers. Concordant with our pyrosequencing data showing no significant methylation changes in SIV controllers, gene expression profiles in HIV controllers were documented as being similar to that of uninfected subjects and different from viremic individuals [66, 67]. The different profiles in DNA methylation between SIV controller animals and viremic MAC could be either a direct consequence of SIV replication in the latter and/or its associated immune activation. However, AGMs also strongly control viral replication in LN and, despite this, showed more methylation changes than SIV controllers in LN. While DNA methylation changes were apparent in nonpathogenic SIVagm infection, these were most often not related to immunological anti-viral response pathways. Strikingly, DNA methylation changes in viremic AGM were different from those detected in viremic MAC. Pathways involved with tissue integrity and immune regulation were enriched in AGM. This could thus contribute to the lack of chronic inflammation and intact tissue structures (such as integrity of follicular dendritic cell network and lack of fibrosis in LN of AGM in contrast to MAC) [10, 68, 69]. It is in agreement with observations that AGMs are able to actively maintain integrity of the gut mucosal barrier throughout SIV infection [69–71]. The lack of tissue damage could help AGM to avoid disease progression as dextran sulfate sodium-induced colitis, and tissue damage during SIVagm infection was demonstrated to lead to increased viral load, microbial translocation and inflammation [72].

Another observation of interest in the AGM was the differential methylation of regulatory genes, such as *LAG3*, *IL21* and *IL31* in AGM. The IL-31 cytokine is known to be mainly secreted by activated CD4 + T cells, often Th2 cells, acting on a broad range of immune and non-immune cells. IL-31 is involved in a variety of fundamental physiological processes, such as cell growth, metabolism and immune regulation [73]. IL-21 is known to be secreted by T_FH_ cells in LN and upregulated in Th17 and Th2 cells. IL-21 is reduced in HIV/SIV infections and used as immunotherapy in preclinical trials in MAC to restore Th17 cells and reduce inflammation in the gut during SIVmac infection [74, 75]. IL-21 is known to stimulate NK and CD8 + T cell anti-tumor response [76]. Therefore, the enrichment in favor of Th2 (and not Th1 as in MAC) concomitant with eventually stronger IL-21 expression compared to MAC might contribute in AGM to the lower inflammation and/or induction of lymphocytes with improved suppressive capacity upon SIVagm infection [77].

To our knowledge, this is the first time that such a genome-wide epigenetic study has been performed in CD4 + cells from tissues longitudinally from baseline throughout a viral infection. Our study revealed that in the animal model of HIV (macaques infected by SIVmac), the differentially methylated sites are more frequent in LN than in blood in both acute and chronic phase of infection. All over, the pattern we detected could contribute to explain why ART initiation even in primary HIV-1 infection does not fully revert the metabolic CD4 + T cells back to normal [20, 21, 78]. Moreover, this study identified targets that are associated with nonpathogenic SIV infection and exploring their potential role in tissue repair or resilience could ultimately contribute to identifying novel targets for host-directed therapies to reduce tissue damage and comorbidities. Finally, some modulated pathways identified in CD4 + T cells here have also been described in inflammatory disorders. For instance, CD4 + T cells in Lupus patients show epigenetic changes in the insulin pathway [79]. Thus, our study might point to pathways commonly impacted by HIV and CD4 + T cell-associated inflammatory disorders.

In conclusion, we show that the infection with SIV led to specific changes in DNA methylation in CD4 + T cells, in particular in the LN and already during primary infection. Moreover, epigenetic changes in genes associated with Th1 cell differentiation and metabolism could be linked to pathogenic infection and were absent in aviremic macaques and natural hosts. Nonpathogenic SIVagm infection was characterized by epigenetic modifications in genes associated with immunoregulation and tissue integrity. These genes could represent novel candidates for immunotherapies targeting pathways associated with non-AIDS-related comorbidity and/or abnormal immune activation of CD4 + T cells.

## Methods

### Sample collection and animal infections

For the DNA methylation microarray cohort, the samples have been collected from 19 AGM (*Chlorocebus sabaeus*) and 16 Chinese rhesus macaques (*Macaca mulatta*). The AGM and Chinese rhesus macaques were infected i.v. with 250 TCID_50_ of SIVagm.sab92018 or 50 AID_50_ SIVmac251, respectively, as described previously [32]. For the pyrosequencing validation cohort, we used samples collected from 17 Indian rhesus macaques, out of which 13 were infected with repeated intra-rectal low doses of 250 TCID_50_ SIVmac251, an oral dose of 6000 TCID_50_ SIVmac239 or an intra-venous dose of 100 or 500 TCID_50_ SIVmac239, resulting in 6 viremic and 7 SIV controllers (Additional file 2: Table 1).

Blood was collected by venipuncture on EDTA tubes and shipped to Institut Pasteur to follow up infection parameters. Blood samples were collected at days − 53, − 49, − 35, − 7, 2, 7, 9, 14, 28, 35, 52 and chronic infection (day 62–day 1787, Additional file 2: Table 1). Peripheral lymph nodes were collected at baseline, day 14 and chronic infection (day 62–day 850, Additional file 2: Table 1). Material from the pyrosequencing cohort was collected at necropsy and cryopreserved.

### CD4 + T cell isolation and DNA extraction

CD4 + T cells from peripheral blood mononuclear cells (PBMC) and LN were purified as described previously [32]. Briefly, surgically removed LN were disrupted mechanically after which CD4 + cells were isolated using magnetic anti-CD4 beads (Miltenyi). CD4 + T cell purity after isolation was confirmed using flow cytometry (median 97%, IQR 93–98%, Additional file 1: Figure 5). DNA was extracted from CD4 + T cells using the DNeasy blood and tissue kit (Qiagen), according to manufacturer’s protocol.

### SIV viremia and DNA quantification

Viral RNA was measured by qPCR as described previously [32]. Viral DNA was measured in CD4 + T cells by qPCR as previously reported, using primers and probes designed specifically for SIVagm.sab and SIVmac [25, 31]. Positive and negative controls were used to exclude sample contamination. Sample preparation, enzyme mix preparation and PCR setup were performed in three separate rooms also to avoid PCR contamination. Viral copy numbers were calculated using plasmids of SIVagm and SIVmac, respectively. Primers and plasmids for *CCR5* were used to normalize the viral levels to the number of cells [80].

### Flow cytometry

Whole blood and LN cells in suspension were stained with the following antibodies: CD95 (DX2), CD3 (SP34-2), CD4 (L200) (all BD Biosciences), CD28 (CD28.2), CD20 (2H7) (both eBioscience) and Ki67 (MIB-1, Dako). FcR Blocking Reagent (Miltenyi) was used to block unspecific antibody binding, and live/dead cell viability dye (Invitrogen) was used to exclude dead cells. Cells were labeled with surface-binding antibodies, then fixed with 4% paraformaldehyde and permeabilized using saponin prior to incubation with Ki-67. Events were collected on a BD LSR-II flow cytometer, running BD FACS Diva 6.0 software, and analyzed with FlowJo 9.4.10 (TreeStar). Anti-mouse compensation beads (BD Biosciences) and Arc Amine Reactive Compensation Bead Kit (Life Technologies) were used to define compensation levels.

### Measurement of insulin sensitivity

Plasma from EDTA tubes collected after overnight fasting was used to determine levels of insulin (ELISA, Merck Millipore) and glucose (Abcam, enzymatic assay) following manufacturer’s instructions. Insulin sensitivity was calculated from these levels using the quantitative insulin sensitivity check index (QUICKI) score: 1/(log(fasting insulin [µU/mL]) + log(fasting glucose [mg/dL])) [81].

### DNA methylation microarrays and DMP calling

DNA methylation was measured at three timepoints for MAC: before infection, during acute (day 14 p.i.) and during early chronic infection (day 62–65 p.i.) (Additional file 1: Figure 3 and Additional file 2: Table 1). For AGM, we measured changes in DNA methylation in CD4 + T cells at the same three timepoints in LN and at two timepoints in blood (before infection and early chronic infection, as not enough material was available during acute infection). Additional pre-infection and chronic infection samples were also analyzed (Additional file 2: Table 1).

Bisulfite treatment was performed on 1 µg of DNA using the EpiTect Fast DNA Bisulfite kit (Qiagen), and 200 ng of bisulfite-converted DNA was hybridized to the Infinium HumanMethylation450K BeadChip according to manufacturer’s instructions.

Since the 450K BeadChip is designed for the analysis of human samples, probes compatible with the *Macaca mulatta* and *Chlorocebus sabaeus* genomes were identified, yielding > 160,000 of the 450 K probes targeting functional CpGs in the respective genomes with a maximum of a single mismatch underlying the probe sequence with the exception of the last 5 bases from the 3′ end. This resulted in the ability to study 162,053 probes for AGM and 158,970 probes for MAC, covering 16,259 and 16,611 genes, respectively [38]. The recently developed annotations files [38] were used for each species, and data were filtered and normalized (AGM and macaques separately) based on the CpG island categories of the species-specific annotation files with the SQN Pipeline [82]. All probes with a detection *p* value above 0.01 were removed. Following this filter, 157,164 and 154,108 probes were detected with high confidence in the current study for AGM and MAC, respectively. A threshold of 90% validated probes compared to the annotation file was set as a sample quality control, leading to the removal of one sample. Intra-individual normalization was performed using a subset quantile normalization approach based on the new functional annotation of probes followed by quantile normalization between samples.

Differentially methylated probes (DMP) were identified for each tissue by first filtering probes with a 5% difference compared to baseline in paired samples, followed by a Mann–Whitney test between baseline and the acute or chronic phase taking into account both paired samples and additional unpaired samples. Probes with a nominal *p* value < 0.005 and a median methylation difference > 5% were considered significant.

### Bisulfite pyrosequencing

Differentially methylated probes of interest were validated using bisulfite pyrosequencing. Primers for regions of interest were designed based on the rheMac8 (UCSC) genome build, and the 1 µg of genomic DNA was bisulfited with EpiTect Fast DNA Bisulfite kit (Qiagen) following manufacturer’s instructions. Bisulfited DNA (1 µl) was amplified by PCR (see Additional file 5: Table 4 for primers sequences) with either Hot Start Taq DNA Polymerase (Qiagen) or Phusion U Hot Start PCR Master Mix (ThermoFisher) at 95 °C for 15 min, followed by 50 cycles at 95 °C for 30 s, 10 s at the respective annealing temperature and 72 °C for 20 s and a final extension step at 72 °C for 5 min. Quantitative DNA methylation analysis was performed by pyrosequencing on a PyroMark Q96 MD (Qiagen) and analyzed using the Pyro-Q-CpG software (Qiagen).

### Transcriptome analysis

Transcriptomic microarray data that we previously published using the same CD4 + T cell purification method, viral inoculations and obtained at the same timepoints were reanalyzed to integrate these data with our methylome data [32]. Probe expression values were extracted using R Bioconductor. Scale normalization was performed, and control probes were removed after normalization. Aggregation of probe expression values was then performed at the gene level using median values. Differentially expressed genes were identified using a two-tailed paired Student *t* test (*q* value < 0.05 and fold change > 1.5). *p* value s were corrected using the FDR method. Individual fold-change expression values were calculated based on their matched control samples. Heatmaps and multidimensional scaling (MDS) were generated using R. The Kruskal stress indicated in the MDS representations indicates the quality of the representation and are proportional to the amount of information lost during the dimensionality reduction process. Functional enrichment analyses were done using ingenuity pathway analysis (IPA, Qiagen).

### Statistics

Statistical inference analyses were performed using Prism 5.0 (GraphPad) and R. The Spearman rank test was used to assess the correlations between variables. Two-tailed nonparametric tests were used throughout to compare between groups.

## Supplementary information

**Additional file 1.** Supplementary figures.

**Additional file 2.** **Supplementary Table 1.** Description of animals used for characterization of DNA methylation during SIV infection.

**Additional file 3.** **Supplementary Table 2.** List of all identified differentially methylated probes (DMP) as defined by the genome-wide array.

**Additional file 4.** **Supplementary Table 3.** List of genes that were both differentially methylated and differentially expressed in AGM and MAC as defined by the genome-wide array.

**Additional file 5.** **Supplementary Table 4.** List of primers used for pyrosequencing.

## Data Availability

DNA methylation microarray data including both raw and processed data (filtered and normalized) have been deposited in GEO (GSE143708). Gene expression data were previously described [32] and are deposited in the transcriptome data repository MACE (https://mace.ihes.fr), under accession numbers 3070984318 (AGM) and 2,932,572,286 (RM). All other underlying data are provided in the manuscript or available upon request.

## Abbreviations

SIV: Simian immunodeficiency virus
PLWH: People living with HIV
AGM: African green monkeys
MAC: Macaques
LN: Lymph nodes
PBMC: Peripheral blood mononuclear cells
DMP: Differentially methylated probes
DMG: Differentially methylated genes
DEG: Differentially expressed genes
p.i.: Post-infection
snoRNA: Small nucleolar RNAs
