# Supplementary material for: DNA methylation changes in metabolic and immune-regulatory pathways in blood and lymph node CD4 + T cells in response to SIV infections
Source: Clin Epigenetics. 2020 Dec 9;12:188. doi: 10.1186/s13148-020-00971-w (PMC7724887; doi:10.1186/s13148-020-00971-w)
Supplement: Supplementary file 1 — Additional file 1. Supplementary figures. [file 13148_2020_971_MOESM1_ESM.pdf]

Supplementary Table 1. Description of animals used for characterization of DNA methylation during SIV infection. RM = Rhesus Macaque, AGM = African green monkey, pLN = peripheral lymph node, mLN = mesenteric lymph node. i.r. = intra-rectal, i.v. = intra-venous. *Microarray quality control data from the sample AGM07199 lymph node showed 89% probes with poor quality and this sample was excluded from analysis.

Supplementary Table 2. List of all identified differentially methylated probes (DMP) as defined by the genome-wide array.

Supplementary Table 3. List of genes that were both differentially methylated and differentially expressed in AGM and MAC as defined by the genome-wide array.

Supplementary Table 4. List of primers used for pyrosequencing.

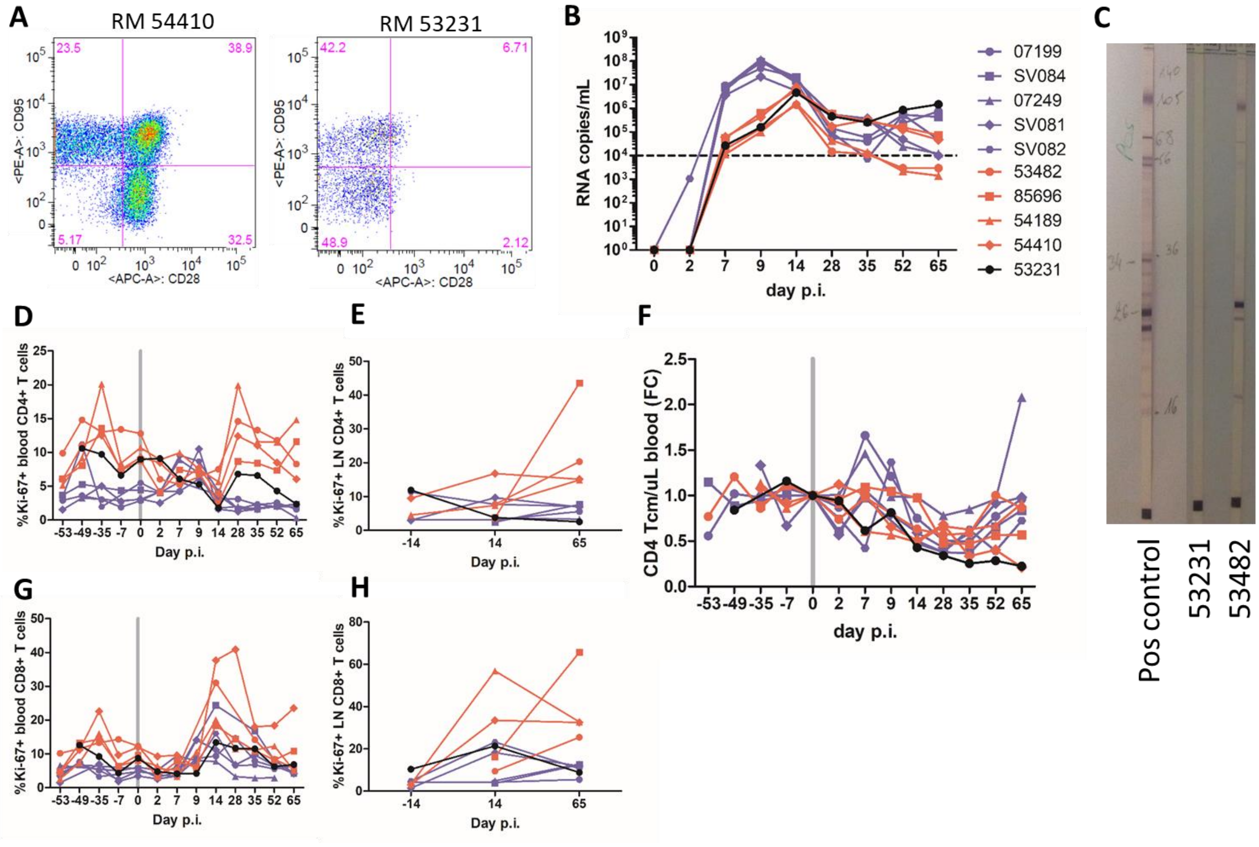

Supplementary Figure 1. Characteristics of the outlier macaque lacking CD28 expression. (A) Dot plots of CD28 expression on CD4+ T cells from PBMC from a macaque (RM 54410) expressing CD28 and macaque RM 53231 lacking CD28 expression. (B) Viremia of African green monkeys (AGM, blue), macaques (MAC, red) and outlier macaque RM 53231 (black). (C) Analysis for anti-HIV antibodies in plasma collected during chronic phase of infection from RM 53231 and an animal with typical seroconversion (RM 53482). Levels of Ki-67+ CD4+ T cells in (D) blood and (E) lymph node. Grey bars indicate the timepoint of SIV infection. (F) CD4+ Tcm cell counts in blood following SIV infection. For RM53231 CD95+ cells were used to define central memory cells due to the absence of detectable CD28 expression. Levels of Ki-67+ CD8+ T cells in (G) blood and (H) lymph node.

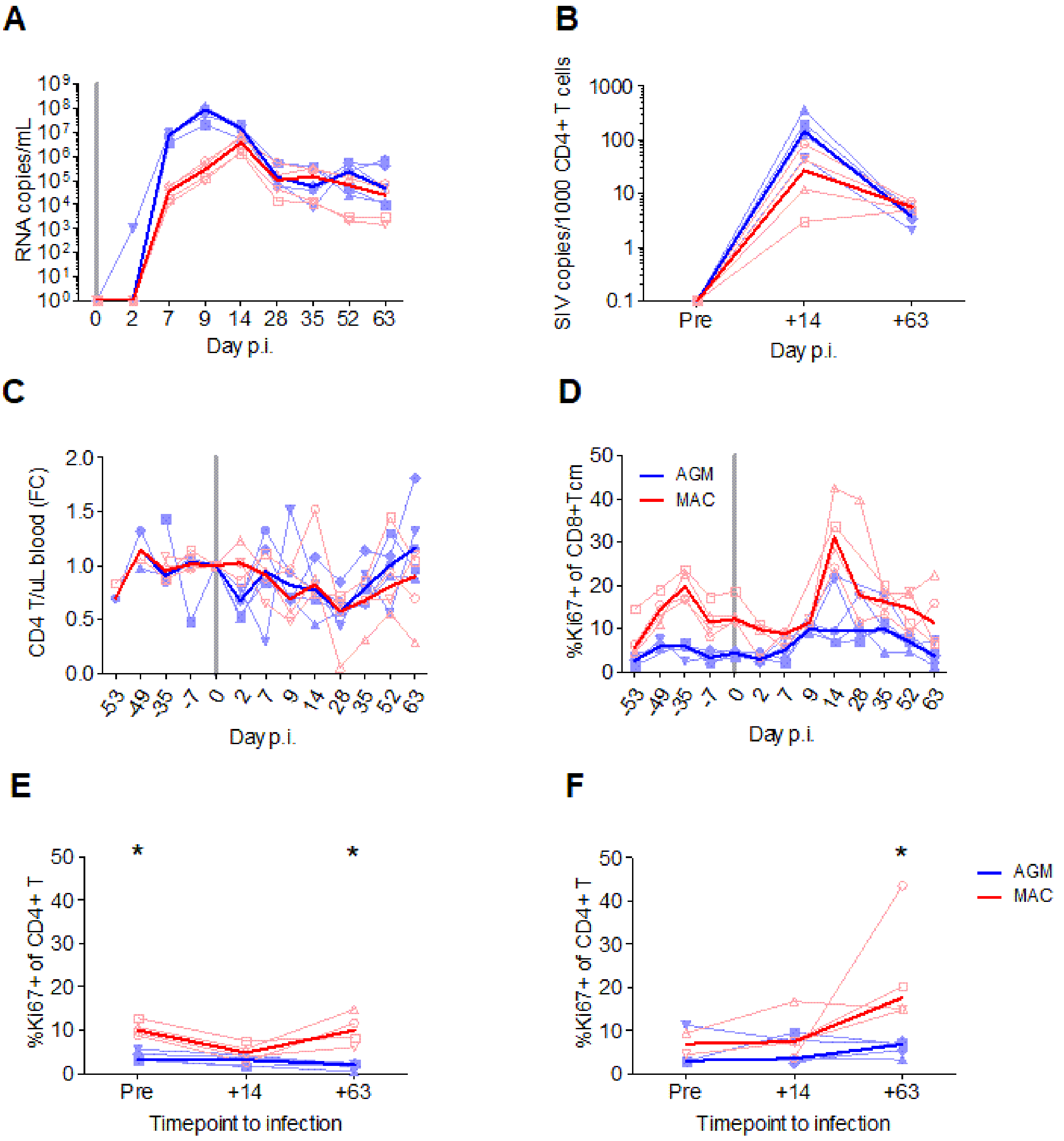

Supplementary Figure 2. Longitudinal follow-up of SIVagm.$_{sab92018}$ and SIVmac$_{251}$ infections for paired methylome analysis. Five AGM (light blue) and four rhesus MAC (light red) were infected with SIV and (A) viremia, (B) Number of SIV DNA copies in CD4+ T cells in LN, (C) CD4+ T cell count fold changes in blood and (D) %Ki-67+ CD8+ T

central memory cells (Tcm) in blood, were followed. AGM showed a lower CD8+ Tcm cell proliferation during the acute phase than the MAC at 9.9% and 31.2% Ki-67+ of CD8+ Tcm cells, respectively. (E) Blood CD4+ T cells displayed increased levels of proliferation in MAC compared to AGM at both baseline and day 63 p.i. (F) MAC LN CD4+ T cells showed increased proliferation compared to AGM in chronic infection (17.7% versus 6.9% Ki67+ CD4+ T cells, $p = 0.016$. Median and individual levels are shown for MAC (individual animals light red, median dark red) and AGM (individual animals light blue, median dark blue, n = 3-5). * $p < 0.05$, Mann-Whitney test comparing AGM and MAC.

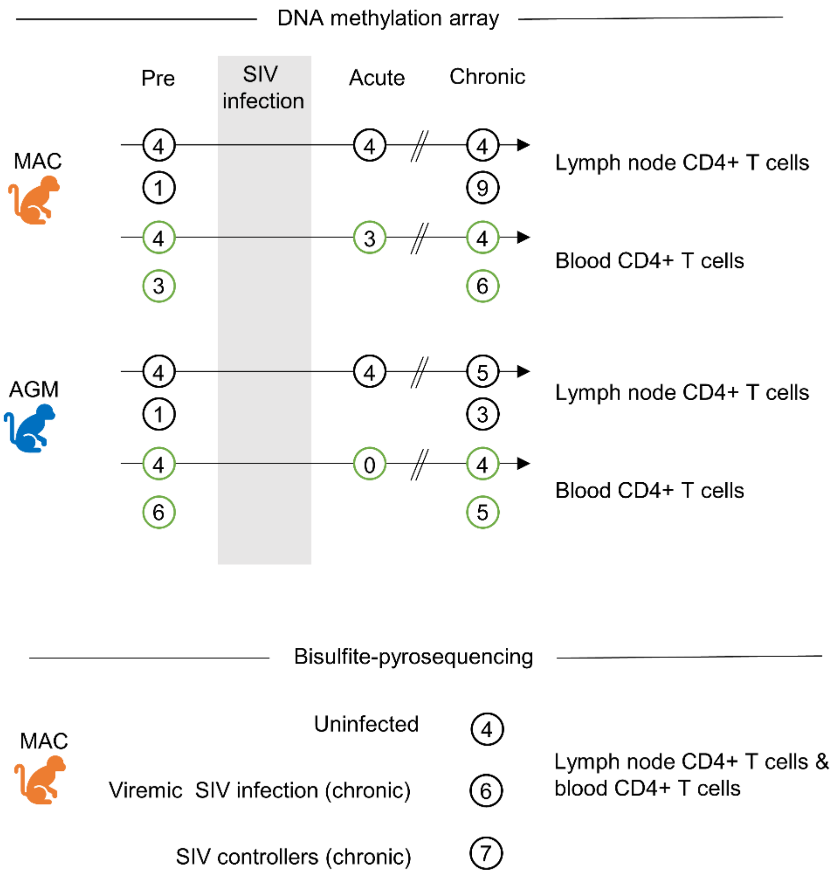

Supplementary Figure 3. Schematic representation of samples collected for DNA methylation analysis by microarray (above) or pyrosequencing (below). Paired samples were collected from 4 MAC and 5 AGM throughout SIV infection, which are connected by a line. The number of animals from which enough DNA of CD4+ T cells were obtained for analysis are indicated per condition. Samples from an additional 12 MAC and 14 AGM at either pre or chronic infection were collected for microarray analysis. Blood and lymph node samples are indicated by green and black circles, respectively. For an additional 17 MAC (uninfected, viremic infected animals and SIV controller animals) lymph node and blood samples were collected for pyrosequencing validation of selected sites. MAC =

macaque. AGM = African green monkey. SIV = simian immunodeficiency virus. See supplementary table 1 for details regarding included animals.

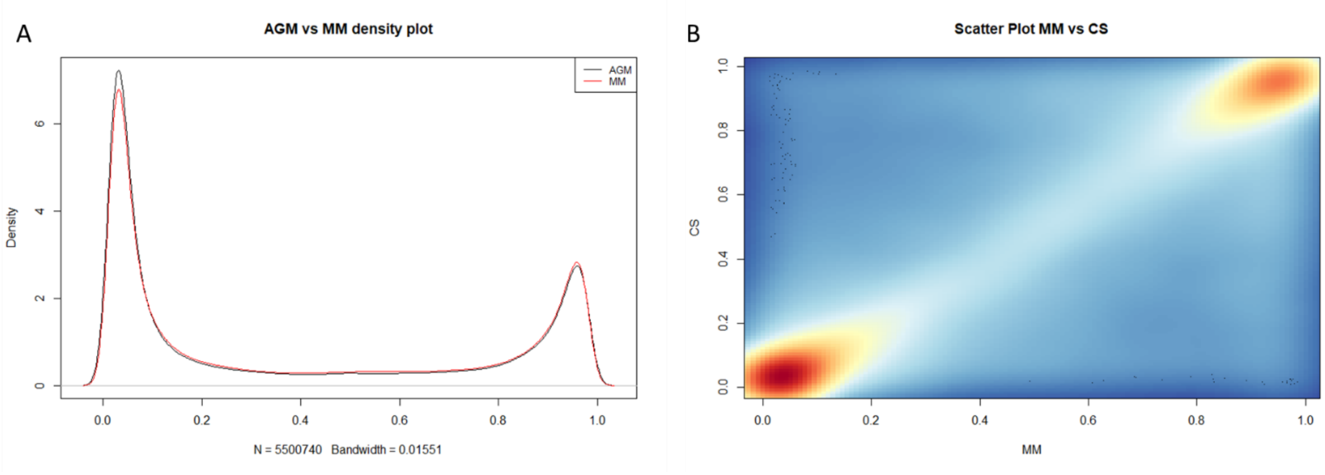

Supplementary Figure 4. Genome-wide methylation patterns in AGM and MAC. A) Density plot showing the distribution of DNA methylation values for AGM (black) and MAC (MM, red). B) Correlation of DNA methylation values between the two species for each of the probes. Color reflects intensity with red increased number and blue a decreased number of probes, Spearman's rho=0.95, $p=2.2*10^{-16}$.

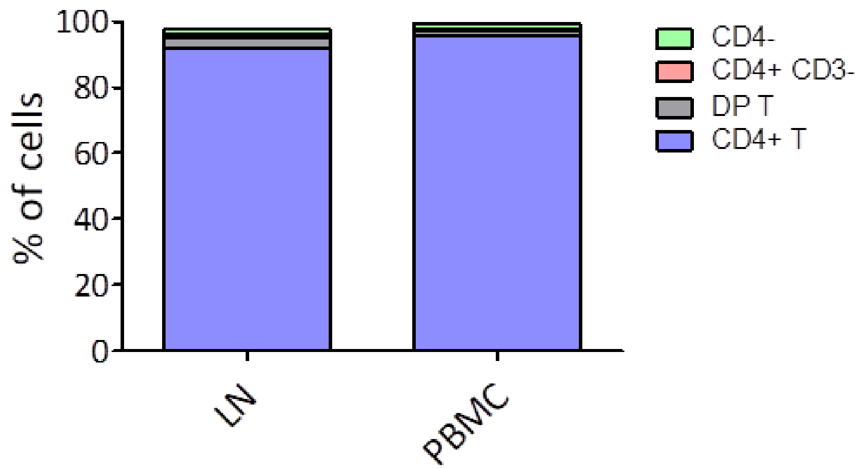

Supplementary Figure 5. Composition of CD4+ T cells purified by column. Median levels are shown for CD4+ T (blue), CD4+ CD8+ double positive T cells (DP T, grey), CD4+ CD3- non-T cells (red), and CD4- cells (green). LN = lymph node, PBMC = peripheral blood mononuclear cells.
